# Screening H3 Histone Acetylation in a Wild Bird, the House Sparrow (*Passer Domesticus*)

**DOI:** 10.1093/iob/obae004

**Published:** 2024-02-27

**Authors:** D Ray, E L Sheldon, C Zimmer, L B Martin, A W Schrey

**Affiliations:** Department of Biology, Georgia Southern University, Savannah, GA 31419, United States; USF Global Health and Infectious Disease Research Center and USF Genomics Center, College of Public Health University of South Florida, Tampa, FL 33612, United States; Laboratoire d'Ethologie Expérimentale et Comparée, LEEC, Université Sorbonne Paris Nord, UR 4443, 93430 Villetaneuse, France; USF Global Health and Infectious Disease Research Center and USF Genomics Center, College of Public Health University of South Florida, Tampa, FL 33612, United States; Department of Biology, Georgia Southern University, Savannah, GA 31419, United States

## Abstract

Epigenetic mechanisms are increasingly understood to have major impacts across ecology. However, one molecular epigenetic mechanism, DNA methylation, currently dominates the literature. A second mechanism, histone modification, is likely important to ecologically relevant phenotypes and thus warrants investigation, especially because molecular interplay between methylation and histone acetylation can strongly affect gene expression. There are a limited number of histone acetylation studies on non-model organisms, yet those that exist show that it can impact gene expression and phenotypic plasticity. Wild birds provide an excellent system to investigate histone acetylation, as free-living individuals must rapidly adjust to environmental change. Here, we screen histone acetylation in the house sparrow (*Passer domesticus*); we studied this species because DNA methylation was important in the spread of this bird globally. This species has one of the broadest geographic distributions in the world, and part of this success is related to the way that it uses methylation to regulate its gene expression. Here, we verify that a commercially available assay that was developed for mammals can be used in house sparrows. We detected high variance in histone acetylation among individuals in both liver and spleen tissue. Further, house sparrows with higher epigenetic potential in the *Toll Like Receptor-4 (TLR-4*) promoter (i.e., CpG content) had higher histone acetylation in liver. Also, there was a negative correlation between histone acetylation in spleen and *TLR-4* expression. In addition to validating a method for measuring histone acetylation in wild songbirds, this study also shows that histone acetylation is related to epigenetic potential and gene expression, adding a new study option for ecological epigenetics.

## Introduction

Epigenetic mechanisms can alter gene expression without variation in underlying DNA sequence (Bossdorf et al. 2008). Of the three molecular epigenetic mechanisms: DNA methylation, histone modification, and chromatin structure, only DNA methylation has received much attention in non-model organisms, especially vertebrates, as histone modification and chromatin structure are more complex and more difficult to screen (Schrey et al. 2014; Sheldon et al. 2018). Also, screening histone modification requires working with proteins, which can require techniques beyond what is commonly available in many genetic-based molecular ecology laboratories. Studies in model organisms increasingly have shown histone modification to be important in a wide array of crucial phenomenon, including the regulation of cellular function (Kojima et al. 2023), the establishment of crucial vertebrate cell types (Rao and LaBonne 2018), and the formation of memory (Peixoto and Abel 2013). For example, histone deacetylase activity is essential in regulation of progenitor cell differentiation (Shen et al. 2005), and histone modification in adrenal tissue affects the thermotolerance of chickens (Zheng et al. 2021). Further, the therapeutic applications of histone deacetylase inhibitors have been demonstrated in several cases (Kojima et al. 2023).

Histones are highly conserved proteins that package DNA into the nucleosome (reviewed by Bartova et al. 2008). The amino acid residues comprising histone tails can be covalently modified by up to 15 different subunits (i.e., methylation, acetylation, phosphorylation, and ubiquitylation). Modifications to histone residues alter the reactivity of that histone-DNA complex and hence the conformation of DNA within the nucleus. Histone acetylation is generally an activating modification, promoting transcription by making the DNA more euchromatin like. By decreasing a histone's affinity for DNA, transcription factor binding sites become more accessible, thus bolstering transcription (Bartova et al. 2008).

There are a limited number of histone acetylation studies on non-model organisms, and those that exist indicate that histone acetylation can have a major impact on gene expression, phenotypic plasticity, and plasticity of life history traits (Reynolds et al. 2016, Choppin et al. 2021). For instance, altered histone acetylation facilitated the shift in physiology of worker ants (*Temnothorax rugatulus*) following the loss of a queen, increasing their fecundity, expression of immune genes, and longevity of the hive (Choppin et al. 2021). Changes in histone acetylation were also associated with pupal diapause (i.e., a period of dormancy that is induced by harsh environmental conditions) in the flesh fly (*Sarcophaga bullata*; Reynolds et al. 2016).

Introduced species provide an excellent system to investigate the role of histone acetylation in variation in gene expression. Introduced individuals must rapidly adjust to new environments, a particularly fraught challenge because introduced populations tend to have low genetic variation and differentiation compared to native populations (Schrey et al. 2011; Liebl et al. 2015; Hanson et al. 2022). Ample evidence exists to support that DNA methylation is important in this process (Liebl et al. 2013; Mounger et al. 2021). Indeed, the house sparrow (*Passer domesticus*) has been successfully introduced throughout the world (Liebl et al. 2015), and DNA methylation is important at the individual level to variation in gene expression (Kilvitis et al. 2019) and at the population level in terms of success in colonizing new areas (Liebl et al. 2013; Sheldon et al. 2018; Swaegers et al. 2023). Partly, these population-level effects arise because individuals possess genomes with different dispositions to be epigenetically modified, a trait termed epigenetic potential (Kilvitis et al. 2017; Sheldon et al. 2023). One form of epigenetic potential that has been estimated is the number of CpG sites in promoters. In past work, we have found that epigenetic potential varies among individuals (Hanson et al. 2020), is related to gene expression levels (Hanson et al. 2021), and is greater in house sparrows collected in more recently introduced areas than native ones (Hanson et al. 2022). We propose that histone modification is also important to introduced house sparrows, given that it has an impact on gene expression and phenotypic plasticity (Reynolds et al. 2016, Choppin et al. 2021).

Here, we screened histone modification in wild-collected house sparrows. Our goal was to facilitate investigations of histone acetylation and encourage the use of additional epigenetic markers beyond DNA methylation in ecological epigenetics. To do that we determined whether (1) histone acetylation can be measured in wild collected house sparrows using a commercially available assay developed for mammals, and (2) how variation in histone acetylation is related to a suit of characteristics in house sparrows that were experimentally exposed to *Salmonella enterica* (Sheldon et al. 2023). We focused on two traits, epigenetic potential of the putative *Toll Like Receptor-4 (TLR-4*) promoter and *TLR-4* expression, both are variable in the individuals screened and differ in response to infection (Sheldon et al. 2023). *TLR-4* is a highly conserved pattern recognition receptor that primarily recognizes Gram-negative lipopolysaccharide, making it one of the first lines of defense against infection (Molteni et al. 2016). *Salmonella*-susceptible mouse strains were found to have mutated *TLR-4* (Leveque et al. 2003), and amino acid variants between susceptible and resistant chicken lines were all found in a domain of *TLR-4* responsible for ligand-recognition (Leveque et al. 2003).

We measured histone acetylation in house sparrows that had been previously collected for a study on the response of individuals to *S. enterica* infection (Sheldon et al. 2023). These experimental samples provide us with the framework, in a controlled setting, to investigate relationships among epigenetic potential, acetylation and gene expression. Our primary focus was on epigenetic potential due to its importance in response to infection in Sheldon et al. (2023) and its relationship with *TLR-4* expression (Hanson et al. 2021).

## Methods

### Samples screened

We screened H3 histone acetylation in paired liver and spleen tissues from adult male house sparrows (*n* = 12) collected in June of 2021 from Tampa, Florida (Table 1) for a study on the relationship between epigenetic potential and response to *S. enterica* infection (as described in Sheldon et al. 2023). Birds were help in captivity, exposed to *S. enterica* on the day of capture and tracked until euthanasia 14 days after exposure (see Sheldon et al. 2023). We screened only adult males with similar body masses (Table 2). We targeted liver and spleen tissues as they are both important in immune function. The liver detects pathogens that enter the body through the gut (Kubes and Jenne 2018), while the spleen filters the blood of pathogens and abnormal cells (Lewis et al. 2019). There is a correlation between host susceptibility to *Salmonella* infection and bacterial load in the liver and spleen (Leveque et al. 2003) and both these tissues were harvested in the previous study (Sheldon et al. 2023). Thus, liver and spleen tissue are ideal tissue for our measurement of histone acetylation post infection.

**Table 1:** House sparrow samples with weight of starting tissue, histone extraction concentration (Conc.), results of three replicates (Rep 1, 2, 3), mean histone acetylation (Mean), standard deviation, and coefficient of variation (CV%)

	Tissue	Conc.	Rep 1	Rep 2	Rep3	Mean		
Bird	(mg)	(mg/ml)	(OD/mg/ml)	(OD/mg/ml)	(OD/mg/ml)	(OD/mg/ml)	Std Dev	CV%
3139-L	9.8	0.44	1.50	1.48	1.28	1.42	0.12	8.4
3155-L	9.6	0.49	1.59	1.79	1.51	1.63	0.14	8.7
3157-L	9.9	0.33	2.00	2.54	1.72	2.08	0.42	20.3
3158-L	12	0.29	1.64	2.50	1.59	1.91	0.51	26.7
3159-L	9.6	0.38	1.81	1.83	1.56	1.73	0.15	8.7
3174-L	25.4	0.71	2.16	1.95	2.08	2.06	0.10	5.0
3179-L	8	0.38	1.77	2.27	2.09	2.05	0.25	12.3
3190-L	6.9	0.14	1.24	1.29	1.43	1.32	0.10	7.4
3191-L	4.8	0.56	0.07	0.10	0.09	0.08	0.01	10.1
3162-L	18.6	0.58	1.28	1.65	1.09	1.34	0.28	21.1
3176-L	22.2	0.32	0.96	1.40	0.87	1.08	0.28	26.2
3187-L	5.1	0.79	0.04	0.11	0.06	0.07	0.03	48.2
3139-S	21.7	0.55	1.88	2.34	0.95	1.72	0.71	41.2
3155-S	3.1	0.57	1.94	2.32	0.93	1.73	0.72	41.5
3157-S	8	0.30	2.70	2.49	1.34	2.18	0.73	33.5
3158-S	0.4	0.80	0.10	0.12	0.04	0.08	0.04	46.6
3159-S	16.5	1.13	0.52	0.50	0.30	0.44	0.12	28.2
3174-S	3.5	0.40	2.21	2.23	1.86	2.10	0.21	10.0
3179-S	0.6	0.19	1.10	1.06	0.63	0.93	0.27	28.5
3190-S	1.9	0.40	2.16	2.03	1.62	1.94	0.28	14.6
3191-S	9.1	0.19	1.00	0.71	0.37	0.69	0.32	45.9
3162-S	10.1	0.32	0.20	0.21	0.21	0.20	0.01	3.0
3176-S	3.5	0.30	1.05	0.89	0.98	0.97	0.08	8.2
3187-S	6.1	0.42	0.90	0.83	0.63	0.79	0.14	17.8

**Table 2:** House sparrow samples with sex, gene expression level of *TLR-4*, epigenetic potential (EP), final recorded mass (Final Mass), average change in mass (Change in Mass), final *Salmonella* burden (SB), average *Salmonella* burden (Average SB), and average change in *Salmonella* burden (Change in SB), *Salmonella* burden is measured in log genome equivalents

Bird	Sex	*TLR-4*	EP	Final Mass (g)	Change in Mass (g)	SB	Average SB	Change in SB
3139	M	2.62	high	27.1	−0.075	2.32	1.59	−0.86
3155	M	1.01	high	25.3	0.700	0.84	1.44	0.51
3157	M	1.02	high	20.5	−0.625	2.28	3.17	0.29
3158	M	9.22	high	21.7	−0.200	3.58	2.11	0.26
3159	M	1.27	high	22.5	−0.425	3.12	1.43	0.12
3174	M	0.77	high	20.4	−0.775	2.79	0.81	0.71
3179	M	1.22	high	24.1	0.100	4.26	1.32	4.26
3190	M	2.38	high	20.6	−0.633	3.15	1.95	0.87
3191	M	2.37	high	21.5	−0.15	2.20	1.16	−0.25
3162	M	14.93	low	24.8	0.025	4.13	1.71	1.03
3176	M	4.53	low	24	−0.05	3.63	1.96	0.24
3187	M	1.95	low	22.1	−0.55	2.15	1.95	0.33

For each individual, we compared H3 acetylation to epigenetic potential of the putative *TLR-4* promoter, categorizing epigenetic potential in individuals as low (7 or fewer CpG sites), or high (8 or more CpG sites) following Sheldon et al. (2023), which estimated epigenetic potential by sequencing 500 bp upstream from the transcription start site. Sheldon et al. (2023) found that epigenetic potential ranged from 6 to 10 CpG sites, with 76.3% of birds having either epigenetic potential of 7 or 8 and 23.7% having epigenetic potential of 6, 9, or 10. Further, Hanson et al. (2021) also quantified epigenetic potential in the putative *TLR-4* promoter of house sparrows finding that 96.7% of birds had epigenetic potential of 7 or 8. In both studies, treating epigenetic potential as a binary variable was the best predictor of *TLR-4* expression in house sparrows.

We compared H3 acetylation to the level of the *TLR-4* gene expression in the cecum estimated by qRT-PCR from RNA extracts using primers targeting the *TLR-4* transcript (Sheldon et al. 2023). We used *TLR-4* expression as an indicator of change in gene expression among individuals in response to infection, in an attempt to link variation in histone acetylation to individual phenotype. Given that our H3 acetylation estimates are genome-wide, and not localized to particular genes, we do not expect there to be a direct causal relationship between H3 acetylation and *TLR-4* expression. Nor do we expect there to be a direct causal relationship between H3 acetylation and EP, as EP was estimated based on the number of CpG sites, which are directly relevant to DNA methylation. However, as histone modification and DNA methylation are epigenetic mechanisms, it may be reasonable to expect that individuals with higher EP for DNA methylation might also have higher EP for histone modification. Our intention in comparing histone acetylation to *TLR-4* expression and EP was to ground the global estimates of H3 acetylation to ecologically relevant factors at the individual level.

In addition, we compared H3 acetylation to a suite of characteristics recorded for the house sparrows over the course of the experiment. These characters were final recorded body mass, average change in body mass over the experiment, final recorded *Salmonella* burden, and average change in *Salmonella* burden over the experiment (Table 2; see Sheldon et al. 2023).

### Measuring histone acetylation

We extracted proteins from tissues using the EpiQuick Total Histone Extraction Kit (EpiGentek, Farmingdale, NY). We then stored the extracts at −80°C overnight, and measured H3 histone acetylation in triplicate the next day using the Total Histone H3 Acetylation Detection Fast Kit (EpiGentek, Farmingdale, NY). Briefly, the Total Histone H3 Acetylation Detection Fast Kit detects H3 acetylation by ELISA. Strip wells are coated with an anti-acetyl histone H3 antibody that captures acetyl histone H3 from the protein extract and allows the experimenter to colorimetrically detect if histone H3 is acetylated, and colorimetrically quantify the amount of the acetylated histone H3. The H3 histone presence is detected and measured using a labeled detection antibody and a color development reagent. We measured the resultant color change with a plate reader (Bio Tek, Synergy HTX Multimode Reader, Agilent, Santa Clara CA. USA).

### Data analysis

We calculated mean H3 acetylation by averaging the replicates and controlling for total protein concentration. We evaluated the repeatability of the raw results by calculating the coefficient of variation (*CV*) among replicates for each individual within tissues. We used the *CV* estimates to define outliers and performed all subsequent tests on a dataset that excluded the most divergent replicate from any replicate sets with *CV* of 25% or higher. We also calculated *CV* among individuals for each tissue, before and after removing outliers. We tested for differences in H3 acetylation mean and variance between liver and spleen by *t-tests* and *f-tests*. We also tested for differences in H3 acetylation levels in both tissues between individuals with high and low epigenetic potential. Additionally, we compared H3 acetylation to *TLR-4* expression, and the suite of characteristics recorded over the course of the experiment, using Pearson's correlation for both liver and spleen tissues.

## Results

We found that the EpiQuick Total Histone Extraction Kit (EpiGentek) and the Total Histone H3 Acetylation Detection Fast Kit (EpiGentek) were effective in house sparrows. Raw estimates of H3 acetylation over three replicates for each individual ranged from 0.01 to 2.08 OD/mg/ml in liver and from 0.08 to 2.18 OD/mg/ml in spleen. *CV* among replicates ranged from 5.0 to 48.2% in liver and 3.0 to 46.6% in spleen. Our criteria of *CV* 25%, or higher, identified 3 outlier estimates in liver and 7 outlier estimates in spleen. The *CV* among replicates was much lower than the *CV* among individuals before and after removing outliers. Among replicates before removing outliers, *CV* was 16.9% for liver and 26.5% for spleen, and *CV* among individuals within tissue was 49.9% for liver and 65.5% for spleen. Among replicates after removing outliers, *CV* was 12.2% for liver and 10.7% for spleen, and among individuals was 51.1% for liver and 66.1% for spleen. There were no correlations between *CV* (among replicates and after removing outliers) and tissue mass (Liver: *r* = −0.09, *P* = 0.38, Spleen: *r* = 0.17, *P* = 0.29), histone concentration (Liver: *r* = 0.30, *P* = 0.16, Spleen: *r* = 0.26, *P* = 0.20), or mean H3 (Liver: *r* = −0.45, *P* = 0.06, Spleen: *r* = −0.03, *P* = 0.46).

We detected high level of variation among individuals when observed estimates of H3 acetylation were standardized by starting concentration (Table 1). Mean H3 histone acetylation in liver was 3.82 OD/mg/ml (σ = 2.58); in spleen, mean H3 histone acetylation was 3.51 OD/mg/ml (σ = 2.45). We failed to detect significant differences between tissues (*t-test P* = 0.38, *f-test* P = 0.87). We caution that based on the high variation observed, these results should not be interpreted as indicating no differences exist in H3 acetylation among tissue, rather that they indicate high variation among individuals.

**Fig. 1:**
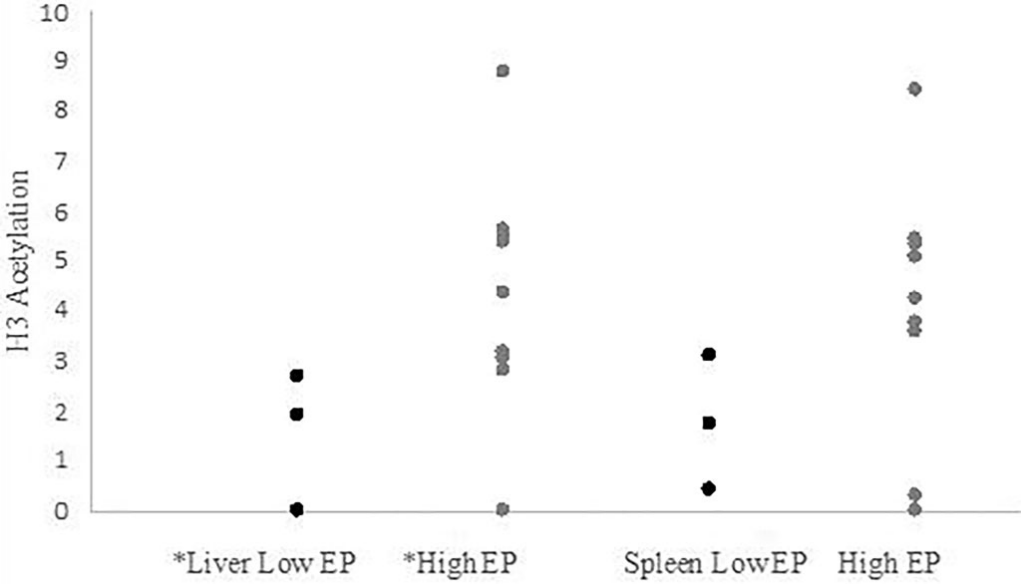
In liver of house sparrows, H3 acetylation differed between individuals with high (Mean H3AC = 4.13) and low (Mean H3AC = 1.56) epigenetic potential (t-test P = 0.046). A similar trend occurred in spleen of house sparrows, high (Mean H3AC = 4.04) versus low (Mean H3AC = 1.78) epigenetic potential, yet was not statistically significant (t-test P = 0.108).

**Fig. 2:**
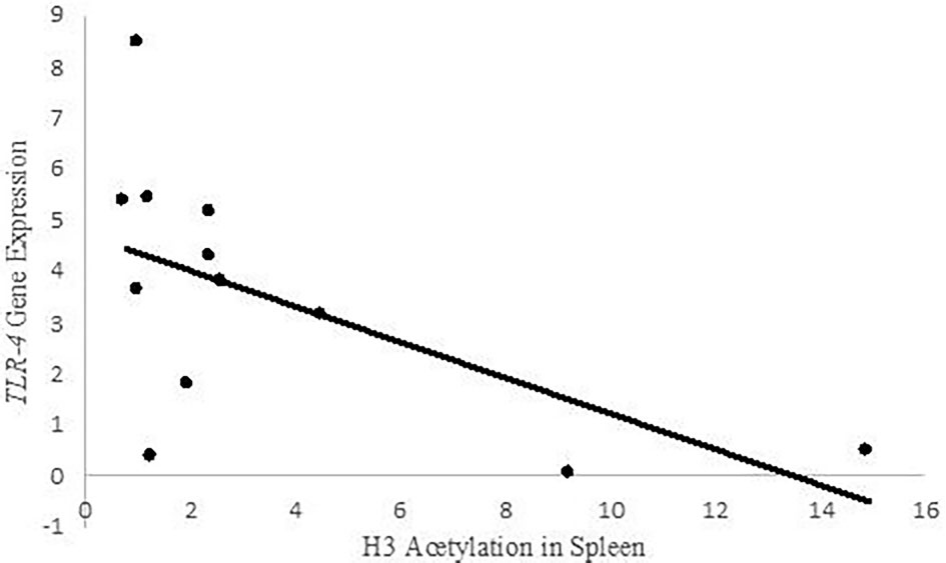
Correlation between H3 acetylation in spleen and TLR-4 gene expression in cecum of infected house sparrows (r = −0.60; P = 0.02).

Interestingly, house sparrows with higher epigenetic potential had higher H3 acetylation in liver tissue (High EP *Mean H3AC* = 4.13 OD/mg/ml; Low EP *Mean H3AC* = 1.56 OD/mg/ml; *t-test* = 0.046), and a similar trend occurred in spleen (High EP *Mean H3AC* = 4.04 OD/mg/ml; Low EP *Mean H3AC* = 1.78 OD/mg/ml; *t-test* = 0.108; Fig. 1). Also, we found a negative correlation between H3 acetylation in spleen and *TLR-4* expression in cecum among house sparrows (*r* = −0.595, *P* = 0.02; Fig. 2).

We failed to detect significant relationships between H3 acetylation and the suite of characteristics recorded over the course of the experimental infection in either liver or spleen. H3 acetylation was not related to final recorded body mass (liver *r* = −0.098, *P* = 0.381; spleen *r* = 0.014, *P* = 0.483), average change in body mass over the experiment (liver *r* = 0.094, *P* = 0.386; spleen *r* = 0.079, *P* = 0.404), final recorded *Salmonella* burden (liver *r* = 0.037, *P* = 0.455; spleen *r* = −0.245, *P* = 0.221), average *Salmonella* burden over the experiment (liver *r* = 0.256, *P* = 0.211; spleen *r* = 0.492, *P* = 0.052), and change in *Salmonella* burden over the experiment (liver *r* = −0.240, *P* = 0.226; spleen *r* = 0.116, *P* = 0.360).

## Discussion

The present study shows that histone H3 acetylation can be screened among wild bird samples using commercially available kits from EpiGentek. We found that H3 acetylation in house sparrows is highly variable. We detected a high *CV* and several outliers (CV above 25%); however, the variation among replicates is much lower than variation among individuals within both tissues, suggesting that the high *CV* values can be attributed to high variation among individuals and not to factors pertaining to the kits. Lower variation within individuals shows that this technique is repeatable, and higher variation among individuals shows that variation exists for selection to act upon. Our study shows that variation exists, and can be effectively screened, making this technique particularly useful. We found no correlations between *CV* and potential sources of error (either technically or biologically). Liver values were more consistent than spleen values, suggesting a difference between tissues. To our knowledge, this is the first study of its kind.

H3 acetylation in house sparrows varies in a manner suggesting that it is an important mechanism to be considered in ecological epigenetics studies. House sparrows with higher epigenetic potential (i.e., more CpG sites in *TLR-4* promoter region) had significantly more H3 acetylation in liver compared to house sparrows with low epigenetic potential. A non-significant, but similar trend also occurred in the spleen. Previous studies on epigenetic potential in house sparrows, based on DNA methylation, found that higher epigenetic potential is important for house sparrows to rapidly adapt to novel environments (Hanson et al. 2021, 2022). It is yet to be determined if estimating epigenetic potential as the number of CpG sites is directly relevant to histone modification, yet our data suggest the two may be related in some manner. At the very least, we expect differences in histone acetylation among individuals with differing epigenetic potential, measured as the number of CpG sites.

We also found a relationship between H3 acetylation and *TLR-4* expression. *TLR-4* is a receptor that detects Gram-negative bacteria and ultimately leads to an inflammatory response (Kilvitis et al. 2019). Histone acetylation being related to *TLR-4* expression suggests that histone acetylation has an effect on gene expression in house sparrows. Our intention for measuring *TLR-4* was to ground the global estimates of H3 acetylation to an ecologically relevant factor at the individual level. Because we measured total acetylation levels, at this time, we cannot be certain exactly how H3 acetylation is associated with gene expression. We recommend caution in interpreting our failure to detect significant relationships between H3 acetylation and the suite of characteristics recorded over the course of the experiment. Of note, we observed a marginally significant relationship between H3 acetylation in the spleen and the average *Salmonella* burden over the course of the experiment (P = 0.052). This trend follows our detecting a significant relationship between H3 acetylation and TLR-4 expression in spleen, but not liver. Taken as a whole, our results support further study of histone modification is an important mechanism in the response of individuals to infection, and more broadly, in the study of invasive species.

For those interested in using EpiGentek's Total Histone H3 Acetylation Detection Fast Kit for ecologically-based studies of birds, we note that, as this kit does not identify specific genomic locations of acetylated H3, the use of experimental designs asking questions relevant to total H3 acetylation levels is critical. For example, asking questions about how different experimental treatments affect total H3 and H4 histone acetylation (Liu et al. 2016). Or, determining if relationships exist between global histone acetylation levels and individual phenotypes (Mosashvilli et al. 2010). Also, we recommend using three replicates and a large sample size, as values were highly variable among individuals.

In conclusion, we describe a fast and inexpensive method for measuring H3 acetylation in birds, and suggest that histone acetylation varies in an ecologically relevant way. Screening histone acetylation will likely add an important new perspective to ecological epigenetics, which has, to date, been dominated by studies of DNA methylation. Given the role that histone modification plays in gene expression and phenotypic plasticity, and its apparent level of individual-level variation, it is very important that we factor this epigenetic mechanism in to future ecological epigenetics studies.

## Data Availability

Data has been submitted to Dryad Total H3 Acetylation in House Sparrows [Dataset]. Dryad. https://doi.org/10.5061/dryad.0k6djhb66.
